# Adverse effects of bedaquiline in patients with extensively drug-resistant tuberculosis

**DOI:** 10.4102/sajid.v35i1.23

**Published:** 2020-10-14

**Authors:** Razia Gaida, Ilse Truter, Charles A. Peters

**Affiliations:** 1 Drug Utilization Research Unit (DURU), Department of Pharmacy, Faculty of Health Sciences, Nelson Mandela University, Port Elizabeth, South Africa; 2 Social Aspects of Public Health Unit, Human Sciences Research Council, Port Elizabeth, South Africa; 3 Jose Pearson TB Hospital, Eastern Cape Department of Health, Port Elizabeth, South Africa

**Keywords:** bedaquiline, adverse effects, extensively drug resistant tuberculosis (XDR-TB), Eastern Cape, World Health Organisation

## Abstract

**Background:**

The World Health Organisation (WHO) guidelines recommend that, because of the resistance patterns of extensively drug-resistant (XDR) tuberculosis (TB) and its unique mechanism of action, bedaquiline be included in the regimen. Although the results of clinical trials have shown bedaquiline to be beneficial, it also carries the risk of adverse effects, some potentially life-threatening. The aim of the study was to determine the incidence of adverse effects caused by bedaquiline in patients diagnosed with XDR-TB. The subsequent management of these adverse effects was also analysed.

**Methods:**

The medical records of patients aged 18 years or older living with XDR-TB who were prescribed bedaquiline in combination with a background regimen at a public-sector drug-resistant TB hospital in the Eastern Cape were reviewed.

**Results:**

Thirty records were reviewed in September 2016. Female patients constituted 66.67% (*n* = 20) of the sample. Nearly half (46.67%; *n* = 14) of the patients were living with human immunodeficiency virus, and six (42.86%) of them were female. Adverse effects were recorded for 26 patients (86.67%) including corrected QT prolongation (40%; *n* = 12), skin rash (33.33%; *n* = 10) and hyperlactataemia (33.33%; *n* = 10) as the most common. There were no treatment discontinuations or deaths. The management of adverse effects varied from omitting doses of bedaquiline to pharmacological intervention.

**Conclusion:**

All patients completed bedaquiline treatment, indicating that the adverse effects did not require discontinuation of the drug. However, when pharmacological intervention is required for the management of adverse effects, care should be taken to ensure that there is minimal interaction with other TB drugs and a low risk of further adverse effects.

## Background

In addition to rifampicin and isoniazid, extensively drug resistant (XDR) tuberculosis (TB) is resistant to both fluoroquinolone (moxifloxacin/levofloxacin) and aminoglycoside (amikacin/kanamycin) injectables.^1^ The World Health Organization (WHO) guidelines^2^ recommend that, because of the resistance patterns of XDR-TB and its unique mechanism of action, bedaquiline be included in the treatment regimen. South Africa has followed the WHO recommendations and advocated that each patient diagnosed with XDR-TB be on a bedaquiline-containing regimen.^3^

Bedaquiline is the first drug to be developed for the treatment of TB in over 40 years.^4,5^ It offers a unique mechanism of action against the mycobacterium by inhibiting the proton pump mycobacterial triphosphate synthase, which is responsible for the production of adenosine triphosphate in the bacterium, subsequently resulting in bacterial cell death.^6,7,8^ Bedaquiline undergoes oxidative metabolism via the cytochrome (CY) P450 pathway, specifically by the isoenzyme CYP3A4, which makes it vulnerable to various drug interactions.^6,7^ Bedaquiline and its primary metabolite, M2, have been shown to have a half-life of approximately five and a half months due to the slow release of these two components from peripheral tissue compartments.^9^

Evidence for the efficacy of bedaquiline comes from two randomised, double-blind, placebo-controlled Phase 2 trials (TMC207 and TMC208) that started in South Africa and expanded to span several countries worldwide.^9,10,11^ The initial study randomised 47 newly diagnosed MDR-TB patients to bedaquiline (*n* = 23), 400 mg daily for 2 weeks, followed by 200 mg three times per week for 6 weeks, or placebo (*n* = 24) in combination with an effective background regimen.^10^ The primary end point was the time to conversion from a positive to a negative sputum culture. The study found that the addition of bedaquiline to the regimen significantly reduced the time to culture conversion and increased the proportion of conversions. The study also noted that adverse effects were mild to moderate in nature, with nausea being the most problematic.^10^ Other adverse effects commonly reported were deafness, arthralgia, haemoptysis, hyperuricemia, pain in the extremities, cutaneous rash and chest pain. These adverse effects were seen in both the bedaquiline and placebo arms and in similar frequencies.^10^ Two years following this initial phase II trial, the same group of patients were reassessed. The study concluded that even though the time to culture conversion was significantly reduced coupled with a higher proportion of patients converting, the number of adverse effects was high; however, these were attributed to the background regimen rather than bedaquiline.^9^ One patient from the bedaquiline group died of a myocardial infarction, although this was not deemed to be due to the bedaquiline itself.

TMC208 was a larger study that randomised 160 patients either to bedaquiline (400 mg daily for 2 weeks, followed by 200 mg three times per week for 22 weeks) or placebo, both in combination with an effective background regimen based on standard of care practices.^11^ The primary end point was the time to sputum culture conversion, defined as two consecutive negative cultures collected at least 25 days apart. Bedaquiline was again found to significantly reduce the time to culture conversion and increase the rate of culture conversion at 24 weeks.^11^ A second follow-up was performed at 120 weeks, and the positive outcomes persisted. Similar rates of adverse effects were found in each group, with the most frequently recorded adverse effects being nausea, arthralgia and vomiting. Ten of the 79 patients (12.7%) randomised to the bedaquiline treatment group and two of the 81 (2.5%) patients randomised to the placebo group died. For six patients (five patients in the bedaquiline group and one in the placebo group), the cause of death was determined to be TB.^11^ None of the deaths were attributed to the use of bedaquiline.

The use of bedaquiline in South Africa was approved by the Medicines Control Council in 2012 through the Expanded Access Programme.^12^ Patients with multidrug-resistant (MDR) TB with limited treatment options could access bedaquiline in South Africa if they met certain criteria. Although the roll-out of bedaquiline was highly anticipated, it is not without its challenges. Bedaquiline is contraindicated in patients who are at high risk of cardiac complications (patients with baseline corrected QT [QTc] interval longer than 500 ms, a history of torsades de pointes or cardiac ventricular arrhythmias or severe coronary artery disease) due to the propensity of bedaquiline to cause prolongation of the QTc interval.^3,6^ A baseline electrocardiogram (ECG) needs to be performed and repeated monthly. If clofazimine or moxifloxacin are being used in addition to the bedaquiline, ECG monitoring needs to be performed weekly during the first month of treatment.^3^ In spite of this warning, no clinical evidence of QTc prolongation has been detected in studies.^10,13^ The follow-up study by Diacon and colleagues^9^ showed that calculating the corrected QT interval using Fridericia’s formula (QTcF; the QTc-interval obtained from the ECG reading is divided by the cubed root of the RR interval obtained from the ECG reading) revealed an increase in the mean QTc interval in both the bedaquiline and placebo groups, but with a higher incidence in the bedaquiline group. However, none of these values were shown to be higher than 500 ms, and no adverse events resulted from these changes. The subsequent introduction of delamanid into the regimen for patients with extensive drug resistance has added to the risk of QTc prolongation when used in combination with bedaquiline, and regular monitoring is imperative.^14^

Hepatic dysfunction is also a concern with bedaquiline, thereby requiring patients to undergo baseline tests and subsequent monitoring throughout the treatment period.^7,15^ Gastrointestinal disturbances such as nausea and diarrhoea have been commonly reported.^9,10,13^ Because of the risk of sudden death reported, bedaquiline should only be used in patients where no alternative regimen can be found.^15^ In spite of this warning, the bedaquiline trials, as mentioned above, did not attribute any of the deaths to the study drug.

While shown to be beneficial, bedaquiline also carries the risk of adverse effects, some more severe than others. However, the trial results and subsequent warnings have been discordant, with uncertainty as to the clinical significance and incidence of these adverse effects, indicating that additional data concerning the safety of bedaquiline is required. The aim of the study was to determine the incidence of adverse effects caused by bedaquiline in patients diagnosed with XDR-TB. The subsequent management of these adverse effects was also analysed.

## Research methods and design

The study design involved a retrospective review of the medical records of patients living with XDR-TB who were prescribed bedaquiline in combination with a background regimen. The study was conducted at a public-sector drug-resistant (DR) TB hospital in the Eastern Cape between September and November 2016. Patients aged 18 years or older who were diagnosed with XDR-TB and prescribed a bedaquiline-containing regimen were included in the study. All the patients included in the study were hospitalised for XDR-TB at the time of the study and prescribed bedaquiline as part of the treatment regimen. The records that were included for analysis were those of the 30 most recently discharged patients from the study site that fit the inclusion criteria.

## Data collection

The data collection tool was self-developed and piloted before data collection commenced. The pharmacy records were consulted, and patients identified to have been prescribed bedaquiline were documented. The corresponding medical records were requested from the archives and selected sequentially, provided that the inclusion criteria were fulfilled. Before bedaquiline was initiated, patients needed to undergo several baseline tests. These included an ECG, liver function tests, a full blood count, glucose levels, lactate and lipase levels and a chest X-ray. Baseline data were obtained from the medical records. The medical records were retrospectively reviewed from the time bedaquiline was initiated until the end of the 24 weeks of treatment in order to determine whether any adverse effects occurred and how they were managed.

## Data analysis

The data were captured in Microsoft Excel and analysed. The data were subject to general descriptive statistics, measures of central tendency (mean, median and mode), frequency distribution and standard deviation.

## Results

**FIGURE 1:**
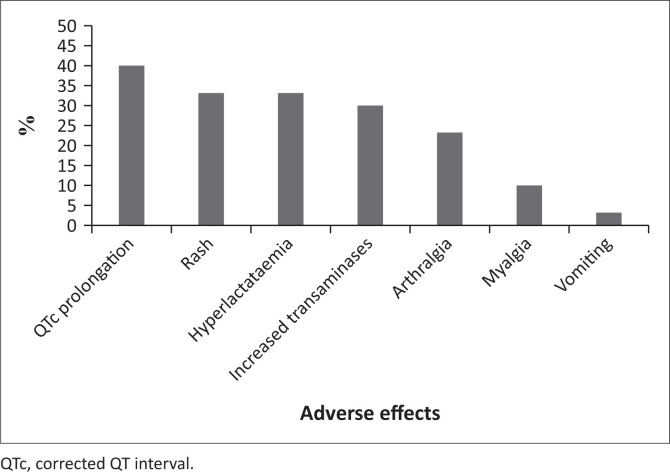
Adverse effects experienced by patients while on bedaquiline treatment (*N* = 30).

A total of 30 records of patients living with XDR-TB were reviewed. The average age of the patients was 31 ± 9.89 years. Female patients constituted 66.67% (*n* = 20) of the total sample. There were 14 (46.67%) patients in the sample living with human immunodeficiency virus (HIV), and eight (57.14%) of these patients were male. All baseline tests were performed for only seven patients. The most common missing results were glucose levels (*n* = 14), lipase levels (*n* = 11), lactate levels (*n* = 6) and chest X-rays (*n* = 6). An ECG was absent for one patient, and a liver function test was absent for one patient. Adverse effects were recorded for 26 (86.67%) patients. All 30 patients completed the 24 weeks of bedaquiline treatment. There were no deaths in the study sample. The adverse effects caused by bedaquiline are outlined in Figure 1.

Corrected QT interval prolongation was the most commonly experienced adverse effect and was recorded for 12 patients (40%), with two of them experiencing persistent QTc prolongation. The QTc was prolonged only while the patient was on bedaquiline. The QTcF was calculated using Fridericia’s formula. The clinical interventions for QTc prolongation are given in Table 1. Half of these patients required no clinical intervention, as the prolongation was not deemed to be clinically significant. The first line of management employed by clinicians at the study site was to omit subsequent doses of bedaquiline in an attempt to normalise the QTc interval. One patient skipped just one dose of bedaquiline and had clofazimine removed from the regimen. Another patient stopped bedaquiline for 1 week (three doses) following the abnormal result and was monitored using ECGs. Neither of these patients had a QTc interval of 500 ms or longer. No patients in the study sample required permanent discontinuation of bedaquiline. Re-challenges were tolerated. Concurrent clofazimine therapy was stopped in three patients, as clofazimine is also known to be associated with QTc prolongation. Other means of management included the prescribing of calcium to two patients, magnesium supplements to four patients and both these elements in combination to two patients.The treatment was empiric, as there were no laboratory results available to confirm deficiency. The adverse effects experienced by patients were therefore not severe enough to warrant the discontinuation of bedaquiline. The WHO^16^ recommends omitting doses of bedaquiline when the QTc is prolonged to normalise the cardiac rhythm. Although bedaquiline has a terminal half-life of five and half months, this action appears to achieve the desired effect because, as seen in the current study, there is no further incidence of QTc prolongation in some cases.

**TABLE 1:** Clinical interventions for corrected QTs interval prolongation.

Patient	Incident	Intervention
Patient 7	1st incident	No intervention
Patient 9	1st incident	Clofazimine stopped
		Magnesium sulphate prescribed
Patient 11	1st incident	One dose of bedaquiline skipped
	2nd incident	No intervention
	3rd incident	No intervention
	4th incident	Clofazimine stopped
Patient 12	1st incident	No intervention
Patient 15	1st incident	No intervention
Patient 16	1st incident	No intervention
Patient 18	1st incident	Repeat ECG in 1 week
Patient 19	1st incident	No intervention
Patient 20	1st incident	Repeat ECG in 24 h
	2nd incident	Blood tests, repeat ECG
	3rd incident	Bedaquiline stopped for 1 week
	4th incident	Monitor
	5th incident	Repeat ECG
	6th incident	Repeat ECG
	7th incident	Repeat ECG
	8th incident	Blood tests, weekly ECGs
	9th incident	No intervention
	10th incident	No intervention
Patient 22	1st incident	Bedaquiline omitted for 1 week
		Magnesium sulphate and calcium carbonate prescribed
Patient 24	1st incident	No intervention
	2nd incident	Clofazimine stopped
		Bedaquiline omitted for 1 week
		Magnesium sulphate and calcium carbonate prescribed
Patient 25	1st incident	Magnesium sulphate prescribed

ECG, electrocardiogram.

Skin rashes caused by bedaquiline therapy were managed using combinations of a systemic antihistamine (chlorpheniramine), topical steroid therapy (betamethasone cream) and a topical moisturiser (aqueous cream). Hyperlactataemia was observed in 10 (33.33%) patients. Three of these patients were prescribed normal saline, to be infused 8-hourly, in order to reduce their lactate levels. Note that this intervention was likely intended as a supportive measure to ensure adequate hydration. The underlying cause of the bedaquiline-related hyperlactataemia would need further investigation for the root cause to be identified and treated. These patients completed bedaquiline treatment. Increased transaminases were detected in nine (30%) patients, but no clinical interventions were made. All ranges were based on those of the National Health Laboratory Service (NHLS). Elevated levels referred to any reading higher than the upper threshold as indicated by NHLS. Clinical interpretation of results was left to the discretion of the attending clinician, and no parameters were set for the purposes of the study. The actual value was unfortunately not recorded for the purposes of the study.

Myalgia and arthralgia were also managed pharmacologically. All patients (*n* = 10; 33.33%) experiencing myalgia or arthralgia were prescribed diclofenac and paracetamol tablets. Whether this treatment provided relief from the pain cannot be said conclusively; however, as no recurring incidences or persistent complaints were recorded, it is possible that the symptoms were relieved. Gastrointestinal disturbances were observed in three (10%) patients in the form of abdominal pain (*n* = 2) and vomiting (*n* = 1). Many of the other TB medications may also have this effect, but as these incidences occurred shortly after bedaquiline was added to the regimen, it is plausible that bedaquiline was the causative agent. Metoclopramide tablets were used to alleviate the vomiting.

**FIGURE 2:**
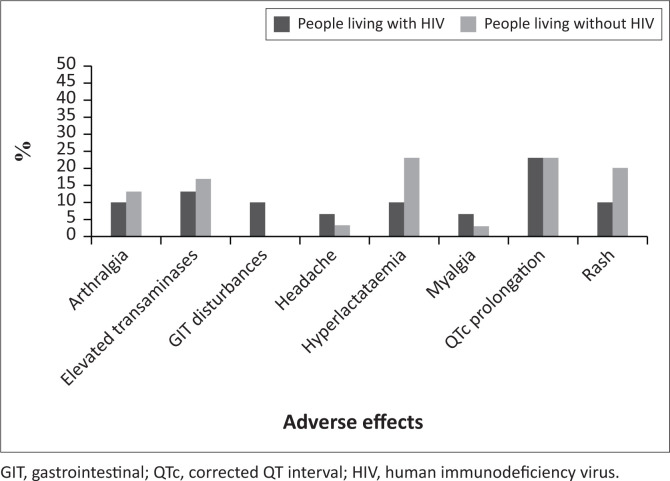
Adverse effects experienced by patients living with and without human immunodeficiency virus being treated with bedaquiline (*N* = 30).

The number of adverse effects experienced by people living with and without HIV were largely similar (30 and 31, respectively) (Figure 2).

Corrected QT interval prolongation dominated the number of adverse effects in both groups, with hyperlactataemia, elevated transaminases and rash being the other commonly experienced challenges.

## Discussion

All 30 patients in the study sample had completed the full 24 weeks of bedaquiline treatment. The adverse effects of bedaquiline experienced by patients were not severe enough to warrant the discontinuation of bedaquiline, and the management of adverse effects was left to the discretion of the clinician at the study site.

Baseline tests are required before the initiation of bedaquiline in order to determine the relative risk to the patient given the known adverse effects of the drug. Not all of the recommended test results were available in the records, indicating that the results were either not recorded, or they were not performed. Keeping in mind that the study site is limited in terms of resources, certain equipment required to carry out certain tests was not always readily available. Examples include glucose and lactate test strips and ECG paper. This is of particular concern as ECG monitoring is essential with the use of bedaquiline. It is imperative that these items always be available and that procurement processes be streamlined. It is recommended that ECG follow-up be done daily according to the USAID/KNCV Tuberculosis Foundation Challenge TB Guide for QTc monitoring and management of drug-resistant TB patients with QT-prolonging agents. If the patient is asymptomatic, follow-up with weekly ECGs until QTc values are normal is recommended by the WHO companion handbook to implementing programmatic bedaquiline.

Corrected QT interval prolongation was the most commonly seen adverse effect associated with bedaquiline. Clofazimine and moxifloxacin both have the potential to cause QTc prolongation;^2^ therefore moxifloxacin is substituted with levofloxacin when a patient is on a bedaquiline-containing regimen. Management strategies included omitting subsequent doses of bedaquiline, discontinuing clofazimine and prescribing magnesium sulphate and calcium carbonate in an attempt to normalise the QTc interval. None of these patients had a QTc interval of 500 ms or longer. These results are in keeping with those of Rustomjee and colleagues^13^ and Diacon and colleagues^9^ in that no clinically significant consequences arose as a result of the prolonged QTc interval. Whilst doses of bedaquiline were omitted in some cases, re-challenges were successful, and all patients completed the 24 weeks of bedaquiline treatment.

Gastrointestinal disturbances and headaches, noted by Diacon and colleagues^9,10^ as common adverse effects, were also seen in 10% (*n* = 3) of the current study sample. Given that many other medications used to treat XDR-TB may also cause gastrointestinal disturbances and that patients may be on additional medication that also has the potential to cause these problems, gastrointestinal disturbances can always be expected and potentially compounded. Arthralgia was reported as a frequent adverse effect by Diacon and colleagues,^11^ reported by 37% of participants, and was also seen frequently in the current sample (33.33%; *n* = 10).

Rashes were a common occurrence in the study sample, affecting 10 (33.33%) of patients. Cutaneous reactions were mentioned as common adverse effects, occurring in 17.39% (*n* = 23) of participants, in the initial Phase II trial conducted by Diacon and colleagues.^10^

It was noted to have occurred in both the placebo and bedaquiline arms, but Rustomjee and colleagues^13^ indicated that the rash was more likely due to bedaquiline as opposed to the background regimen. However, whilst a noted adverse effect of bedaquiline treatment, it cannot be confirmed that skin rashes were due to bedaquiline in the current study.

Increased transminases were noted in the Phase IIb trial conducted by Diacon and colleagues,^11^ but although these increases were higher in the bedaquiline arm as compared to placebo, only three patients had to discontinue the bedaquiline. Two of these three patients had a concurrent hepatitis B infection. Increased levels of alanine and aspartate did not appear to be of clinical significance. No interventions were made, and bedaquiline was not discontinued.

Where there was need for management of adverse effects, the interventions were pharmacological or temporarily withholding the bedaquiline. Adverse effects that require or result in additional pharmacotherapy results in gross polypharmacy, particularly in XDR-TB patients, where the TB regimen in itself is a heavy pill burden. It should be noted that this may influence the adherence to medication on the part of the patient, keeping in mind that even medications used to alleviate the adverse effects of bedaquiline and other anti-TB agents may also cause adverse effects of their own. Patients need to be counselled in such situations and the additional medication stopped as soon as adverse effects have been alleviated. Given the benefits of adding bedaquiline to the regimen, such as earlier and sustained culture conversion^17^ and reduced mortality,^18^ attempts should be made to mitigate or treat adverse effects as soon as possible in order to ensure that treatment is completed to improve patient outcomes.

## Conclusion

In spite of the adverse effects noted, all 30 patients completed the 24-week treatment period with bedaquiline, which indicates that the adverse effects did not require discontinuation of the drug. The management of adverse effects associated with the treatment of bedaquiline is at the discretion of the medical team. However, when pharmacological intervention is required for the management of adverse effects such as arthralgia, rashes or gastrointestinal disturbances, care has to be taken to ensure that there is minimal interaction with other TB drugs and a low risk of further adverse effects. Patients must be counselled on the potential for adverse effects to occur and what to expect with bedaquiline treatment in addition to management therapy when required, as it may influence adherence to the prescribed medication.
